# Plasticised Regenerated Silk/Gold Nanorods Hybrids as Sealant and Bio-Piezoelectric Materials

**DOI:** 10.3390/nano10010179

**Published:** 2020-01-20

**Authors:** Silvia Bittolo Bon, Michele Rapi, Riccardo Coletta, Antonino Morabito, Luca Valentini

**Affiliations:** 1 Dipartimento di Ingegneria Civile e Ambientale, Università degli Studi di Perugia, UdR INSTM, Strada di Pentima 4, 05100 Terni, Italy; silvia.bittolo@gmail.com; 2 Università degli Studi di Firenze Laurea Magistrale in Medicina e Chirurgia, Piazza San Marco 4, 50121 Firenze, Italy; michele.rapi@stud.unifi.it; 3 Department of Pediatric Surgery, Meyer Children’s Hospital, Viale Pieraccini 24, 50139 Firenze, Italy; riccardo.coletta@meyer.it (R.C.); antonino.morabito@unifi.it (A.M.); 4 Dipartimento Neuroscienze, Psicologia, Area del Farmaco e della Salute del Bambino NEUROFARBA, Università degli Studi di Firenze, Viale Pieraccini 6, 50121 Firenze, Italy

**Keywords:** plasticised regenerated silk, mechanical properties, gold nanorods, anastomosis, bio-piezoelectric materials

## Abstract

Manual and mechanical suturing are currently the gold standard for bowel anastomosis. If tissue approximation fails, anastomotic leaks occur. Anastomotic leaks may have catastrophic consequences. The development of a fully absorbable, biocompatible sealant material based on a bio-ink silk fibroin can reduce the chance of anastomotic leaks. We have produced a Ca-modified plasticised regenerated silk (RS) with gold nanorods sealant. This sealant was applied to anastomosed porcine intestine. Water absorption from wet tissue substrate applied compressive strains on hybrid RS films. This compression results in a sealant effect on anastomosis. The increased toughness of the hybrid plasticised RS resulted in the designing of a bio-film with superior elongation at break (i.e., ≈200%) and bursting pressure. We have also reported structure-dependent piezoelectricity of the RS film that shows a piezoelectric effect out of the plane. We hope that in the future, bowel anastomosis can be simplified by providing a multifunctional bio-film that makes feasible the mechanical tissue joint without the need for specific tools and could be used in piezoelectric sealant heads.

## 1. Introduction

Tissue approximation during surgical procedures can be obtained by suturing or stapling [1,2,3,4]. Anastomotic leak is the most concerning complication as it can cause serious infection and need for further surgery [1,2,3,4]. Adhesive sealant creating chemical interaction with the substrate by the diffusion of molecules to the tissue, has been described. Unfortunately, most of the available sealants have poor adhesion or biocompatibility [5,6,7,8].

The available sealant for tissues such as intestine or epidermis, have a complex geometry, are wet and not malleable. This complexity makes the adhesion of the sealant to the tissues difficult. Research on both biocompatible and highly stretchable sealants is challenging due to the lack of available engineered biomaterials.

Previous studies have shown that the fibroin regenerated from the silkworm obtains scaffolds with high neuronal network regenerative power [9,10,11]. The silkworm thread, thanks to its architecture, can be used for studies of intestinal tissue regeneration. We have adopted the dry silk fibroin. It is known that silkworm silk and silk fibroin are biopolymers that are both biocompatible and bioabsorbable.

They are commonly used for scaffolds in tissue engineering [12,13]. We have engineered Ca-modified RS which is able to absorb water and form adhesions on wet tissue. The Ca-modified RS is a material with a high water absorption [14]. The material applies compressive strains at the interface with the substrate, which results in a sealant effect on anastomosed tissues. We have taken inspiration from self-adhesive materials produced in nature by living organisms. Such organisms have the capability to stick on wet interfaces (for example, mussel and spider-web glues) by removing water from the contact surfaces. Gold (Au) nanoparticles were thought to be stable in biologic environments but it has been recently demonstrated that they can undergo intracellular biodegradation and recrystallisation, with a faster degradation of the smallest size particle [15]. Gold nanoparticles maintain stability on solid silk fibroin and promote the dissolution of degummed silk fibres into silk fibroin in a certain CaCl_2_ composition [16].

Regenerated silk (RS), is biocompatible and biodegradable. Regenerated Silk is also a functional material [17,18]. Piezoelectricity is a physical phenomenon that converts mechanical deformation into electricity and vice versa [19]. Thus, materials can be designed to be interfaced with soft tissues and monitor biological forces [20] or can generate electricity from mechanical deformation and used as self-powered force sensors. Unfortunately, the most commonly used piezoelectric materials such as lead zirconate titanate and polyvinylidene difluoride cannot be implanted into the human body due to their intrinsic toxicity or nonbiodegradable structure.

The repeating sequences of amino acid organised in a helical or crystalline sheet structure, connected with each other by intra- and intermolecular hydrogen bonding, are electrical dipoles [21,22]. Thus, regenerated silk could be used as bio-piezoelectric material. The electrical signal generated by applied stress can stimulate the signalling pathways and thereby enhance the tissue regeneration at the impaired site.

We have designed hybrid calcium—modified silk fibroin with multifunctional properties such as optical transparency, stretchability and water responsive shrinking suitable for practical applications in intestinal anastomosis. We have also observed the piezoelectricity of these hybrid films.

## 2. Materials and Methods

### 2.1. Materials Preparation

*B. mori* cocoon were degummed by boiling in NaHCO_3_ (Sigma-Aldrich, St. Louis, MO, USA) solution, at 100 °C for 30 min. The degummed silk fibres were rinsed in deionized water to remove residual NaHCO_3_ and left to evaporate in dry air at room temperature. The dry degummed silk fibres were dissolved in CaCl_2_/formic acid (FA) solution to produce silk solution as already reported [18,19]. Solutions with different SF: CaCl_2_ weight ratios (i.e., 90:10, 80:20 and 70:30) were prepared. To produce 70:30 RS weight ratio, degummed silk fibers (0.4 g) were added to a solution of CaCl_2_ (0.17 g; Sigma-Aldrich) and FA (5 mL; Sigma-Aldrich). The solution was stirred for 30 min at room temperature. The solution was deposited onto a polystyrene dish (50 mm diameter) at room temperature for 8–12 h until FA solvent evaporates the solution was then heated at 60 °C for 2 h to remove the residual solvent. The resulting films were approximately 80 μm thick. Gold nanorods (GNR) colloidal suspension stabilised in cetyltrimethylammonium bromide (CTAB) (diameter × length, 10 nm × 38 nm, ±10%) were purchased from Sigma-Aldrich. CTAB-coated GNR were used to prevent cytotoxicity and cell death due to their superior biological properties, i.e., better biocompatibility and minimal cytotoxicity [23]. One mL of GNR colloidal suspension was added to 5 mL RS solution and mixed at 6 °C to prepare RS-GNR films. The mixture was cast into 50 mm diameter petri dish, dried for 12 h at room temperature and heated at 60 °C for 2 h to remove the residual solvent.

### 2.2. Characterizations

Field-emission scanning electron microscopy (FESEM, Zeiss Supra 25, Oberkochen, Germany) was used to investigate the surface morphology and the cross sections of the samples obtained by fracture in liquid nitrogen. Fourier transform infrared (FTIR, Jasco FT/IR 615, Oklahoma City, OK, USA) analysis was performed in ATR mode in the amide I and amide II regions from 1750 to 1450 cm^−1^. The spectra were deconvoluted by firstly smoothing the signal with a polynomial function with a 15-point Savitski—Golay smoothing function, subtracting a linear baseline and applying a Gaussian deconvoluting curves by Origin 9 software. Ultraviolet-visible (UV-vis) measurements of the films were carried out with a PerkinElmer spectrometer Lambda 35. The mechanical properties of the RS films were measured with a tensile testing machine (Lloyd Instr. LR30K, West Sussex, UK) equipped with a 500 N static load cell. The samples were stretched against their axis at a low strain rate (5 mm/min). Three rectangular shape samples (1.5 cm × 3 cm × 80 µm) for each composition were tested. The Young’s modulus was calculated using the first 0.2% strain region. Tensile strength was defined as the peak stress. Toughness was calculated as the area under the stress–strain curve (see Supporting Information section).

A Porcine intestine loop was sectioned with a scalpel. The two loop cuts created were then anastomosed with 5/0 absorbable interrupted full thickness sutures the suture used is synthetic absorbable monofilament made from the polyester (p-dioxanone).

The anastomosis was completed and the sealant at various strength was applied over the anastomosis. The control intestine did not have any sealant. The intestinal porcine was inflated with water until bursting occurs. A customised digital manometer, connected in series to a peristaltic pump, recorded the bursting pressure, defined as the maximum pressure reached followed by a sharp loss in pressure (see Supporting Information section). The pump filled the line before the insertion into the intestine and the zero was set on the manometer. The intestine section was clamped at both ends and filled with water at a flow rate of 50 mL·min^−1^. The mean value and standard deviation were obtained in 5 sutured intestine portions in each film composition.

For the fabrication of piezoelectric device, the films were stick onto a bottom carbon tape electrode. After that Cu wires were added to the bottom electrode using Ag paste. Then carbon tape was attached to the top side of the film. Due to the presence of film the short between the electrodes was prevented. After that, Cu wires were attached to the top side of the carbon tape (top electrode) using Ag paste.

The device was then encapsulated by crystal liquid rubber (CRISTAL RUBBER purchased from PROCHIMA) that was produced by cold curing the liquid rubber mixed with 10 wt% of PT-CURE catalyst (purchased from PROCHIMA). Silicone packaging is used to protect the device from mechanical stress, humidity and temperature. An external force was applied for the measurement of the piezoelectric output of the devices. The device (up to 500 g, contact area 20 mm^2^ at 1 Hz and 0.2 Hz) and the open circuit voltage as well as the short circuit current values were monitored using a computer controlled Keithley 4200 Source Measure Unit.

## 3. Results and Discussion

### 3.1. Morphology, Structure and Mechanical Properties of the Films

**Figure 1:**
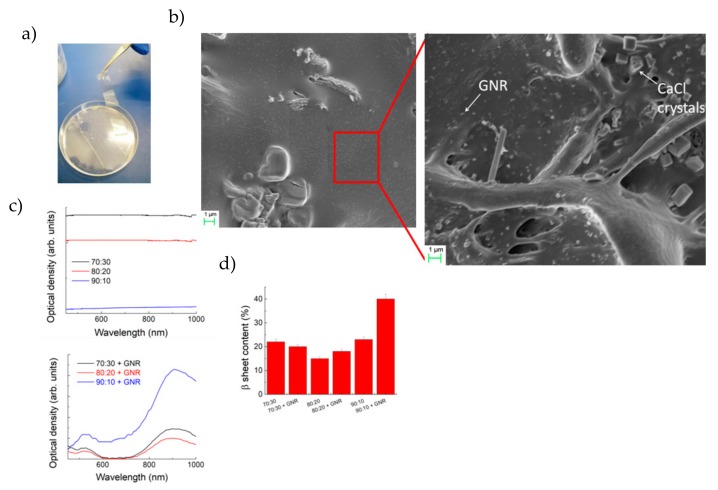
(**a**) Photograph of transparent 70:30 regenerated silk (RS)- gold nanorods (GNR) film and (**b**) FESEM image of 70:30 RS-GNR film. In the magnified image the GNR are indicated by the arrow and their size is compatible with that reported in Figure S1. (**c**) Ultraviolet-visible (UV-Vis) absorbance spectra and (**d**) β-sheets content of RS and RS-GNR films, respectively.

The described method allowed us to produce transparent large films (Figure 1a) with RS and RS-GNR. They were formed as 10 cm diameter and ≈80 μm thick films (see Supporting Information, Figure S1). The 70:30 Ca-modified RS maintained a transparency over 95% (Figure 1a,c). Colloidal dispersions of GNRs onto RS film, 38 nm length and 10 nm width (Figure 1b and Figure S1), showed the characteristic plasmonic resonance peak at 850 nm (Figure 1c), consistent with previous results reported in the literature [24]. Changes in the structure of the films were then investigated by FTIR (Figure 1d). The β-sheet (crystalline domains) content was determined by the deconvolution of the amide I region (1580–1720 cm^−1^). We estimated the ratio between the peak area in the wavenumber region of 1622–1637 cm^−1^, which is the main absorbance region of β-sheet crystal in amide I [25], and the whole area of the amide I (i.e., the peaks of the structural components including turns (T) and random coil (R) reported in Figure S2). The deconvolution of the amide I band provides an estimation of β-sheet structure in the RS and RS-GNR films that is reported in Figure 1d.

**Figure 2:**
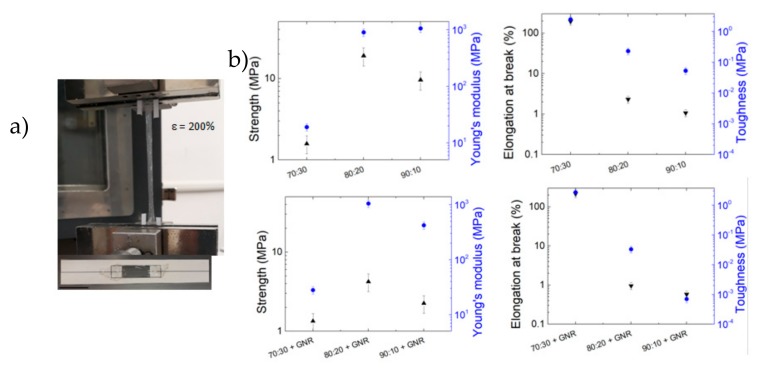
(**a**) Photograph of the 70:30 RS-GNR film glued on rectangular paper frame (scale bar at the bottom indicates 3 cm) and stretched 3 times the original length. (**b**) Effect of Ca content and GNR addition on strength, modulus, elongation at break and toughness of the RS films.

The plasticised RS-GNR when glued on a rectangular planar frame, exhibits the ability to stretch to more than 3 times the original length (Figure 2a). The control of plasticisation is a critical issue for the realisation of stretchable materials. The RS contains Ca^2+^ ions that can capture the humidity from the environment; thus, more calcium ions are in RS, much more stretchable will be the film [14]. Figure 2b shows how the increase of the CaCl_2_ in the RS solution results in increased elongation at break from about 1% to about 200% when the RS/CaCl weight ratio passed from 90:10 to 70:30, respectively. Plasticisation and crystallisation are two key aspects of the secondary structure elements of silk (i.e., β-sheets content). We found that the mechanical strength and Young’s modulus were not correlated with the β-sheets content (Figure 1d and Figure 2b). At 80:20 and 90:10 weight ratios, the RS films stiffen drastically. The low crystalline fraction of our samples is similar to PVA hydrogels with low crystallinity and high water content that, recently [26], have demonstrated to have high tensile strength. Zhao et al. [26], proposed that the combinational properties of stiffness and high water content are due to the co-existence of two separated phases: chains cross-linked by nanocrystalline domains dispersed into swollen amorphous chains.

In Figure 2b we have represented the mechanical results obtained from testing of RS-GNR samples, this will help to demonstrate its potential application of GNR in RS. The maximum average toughness (i.e., the area underlying the stress-strain curves, see Supporting Information, Figure S3) obtained from 70:30 RS-GNR sample was 2.73 MPa and the highest elongation at break obtained was 220%. The presence of GNR can thus plasticise regenerated silk and increase the ultimate strain, leading to the highest toughness value.

**Figure 3:**
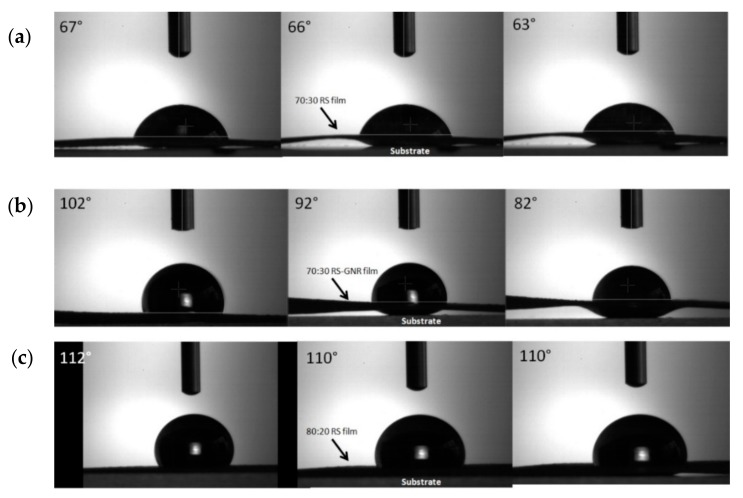
Snapshots of contact angle measurements of (**a**) 70:30 RS, (**b**) 70:30 RS-GNR and (**c**) 80:20 RS films, recorded (from left to right) after 5 s, 10 s and 25 s, respectively. The arrows indicate the contraction of the film moving from the substrate (see Supporting Information, Videos S1, S2 and S3).

While designing RS-GNR film for high stretchability and toughness, we discovered that water capturing of Ca ions created when dissolving CaCl_2_ in solution, led to a physical shrinkage of RS. Contact angle tests (Figure 3) show that for the highest CaCl_2_ content, the RS shows a more hydrophilic surface. This facilitates water absorption and a quick contraction of the film from the substrate, for less than 30 s (Figure 3a,b). By increasing the RS fraction, this effect disappears and a less hydrophilic surface was obtained (Figure 3c).

This shrinking effect is very similar to a well-known property of spider silk called super-contraction, in which the silk fibres can suddenly shrink in response to changes in moisture [27]. Spider silk is a protein fibre like silkworm silk. When water molecules interact with it, they disrupt its hydrogen bonds in an asymmetrical way that causes the contraction at high percent of relative humidity.

### 3.2. Anastomosis Results and Device Fabrication

**Figure 4:**
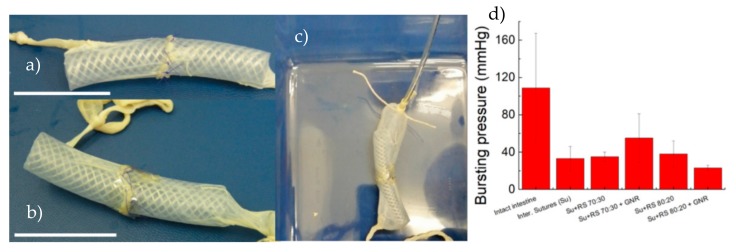
Photographs of (**a**) sutured porcine intestine to determine the anastomosis bursting pressure and (**b**) RS film placed over the suture. The scale bars indicate 5 cm. (**c**) Photograph showing the intestine while is inflating with water and its dilatation until rupture. (**d**) Bursting pressures values obtained from rupture of intact intestine, interrupted sutured (Su) intestine, RS and RS-GNR films on sutured intestine, respectively.

We hypothesised that the inclusion of Ca ions could tune the contractile behaviour of the RS films and program the strain in tissue approximation. Manual suturing is critical for bowel approximation; anastomosis failure can lead to anastomotic leaks, severe infection, further surgery, prolonged hospital stays and in some cases death [28,29,30,31]. Porcine intestine was sectioned, anastomosed and inflated with water while recording burst pressure (Figure 4a,b), which is an indicator of the suture strength [32,33]. Intestinal anastomosis with interrupted sutures, withstands low pressure before bursting (i.e., 40 mmHg). The use of RS and RS-GNR films over the sutured intestine line did not significantly improve the burst pressure. Although 70:30 RS, 80:20 RS and 80:20 RS-GNR films physically well adhered on sutured tissue, they provide only a tenuous barrier to fluid flow. In the case of 90:10 RS and 90:10 RS-GNR, it was not possible to apply the film on the anastomosis due to their mechanical brittleness that hinders the adhesion on curved and irregular tissue. Use of 70:30 RS-GNR over the suture improves burst pressure (i.e., 74 mmHg) compared to that recorded for sealant-free sutured intestine. Therefore, the use of GNR modified RS is effective for providing a barrier to the fluid. Future in vivo experiments are planned.

**Figure 5:**
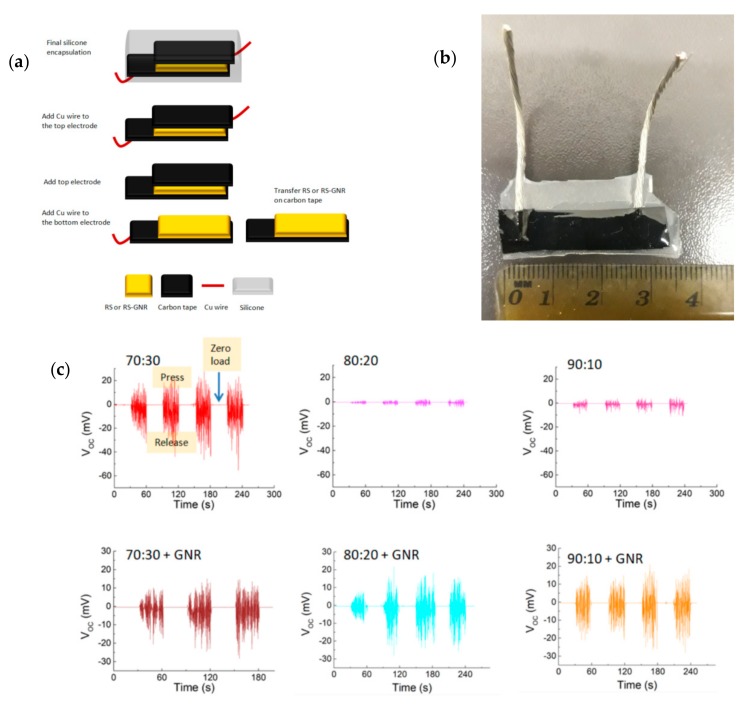
(**a**) Schematic of fabrication of piezoelectric device. (**b**) Photograph of piezoelectric device fabricated. (**c**) The generated output voltage of piezoelectric devices fabricated with different RS and RS-GNR films and subjected to repetitive loading and releasing pressure.

Finally, we realised a piezoelectric device (Figure 5a,b) and investigated the piezoelectricity on our films. Recently, for spider silk, it was shown that silk fibres exhibit a piezoelectric response under external force applied out of plane to the fibres’ axis [34,35,36,37,38]. The authors explained the observed effect with the rubbing between the crystalline domains and the strained elastic helical regions which may lead to developing intermolecular—CONH hydrogen bonding [21]. Thus, the deformation of such elastic amorphous structure may develop high electrical dipoles.

When we dissolved CaCl_2_ in the silk fibroin solution, we introduced Ca ions in the RS. The water capturing of Ca ions enriches Ca-modified silk film of random coil regions, metal chelation [38] and the crystalline domains are disrupted. Furthermore, in analogy to what was observed in spider silk, the dipole orientation inside our films is possible. Application of mechanical pressure on the films should generate separation of positive and negative charges on the top and bottom electrodes, developing potential mismatch between two electrodes for electron transfer to an external circuit. Removing the mechanical pressure from the device, accumulated electrons will move to other electrodes in a reverse direction through an external circuit; thus, we observe a reverse signal.

In Figure 5c we have shown that applying and releasing a load of 5 N (i.e., compressive stress ≈250 kPa) we observed output voltage (V_OC_) and output current (see Supporting Information, Figure S4). According to the previous studies on piezoelectricity of spider silk and the proposed working mechanism [21], we confirmed that the devices made of RS and RS-GNR films with the highest CaCl_2_ content (i.e., the lowest crystalline domains) showed the best performance. Despite the presence of metallic nanorods which act as charge trappers, the output voltage values measured for the RS-GNR films while are slightly lower than that of the 70:30 series instead are higher than those of the 80:20 and 90:10 concentrations. If compared with the most recent performances of bio-based nanogenerators [22], the generated output performance of our films is comparable to those reported in the literature [22] and even higher [21] if normalised the data for the load applied to our device.

The results obtained in this section can be linked to the mechanical and sealant properties for paving the way to the development of pressure sensors that are nowadays required to monitor biological parameters such lung pressure, eye pressure, etc., and have to be implanted directly on biological soft tissues and organs. Therefore, the devices should be flexible, stimuli responsive and biodegradable to avoid subsequent removal surgery, which could be invasive and stressful for some patients.

## 4. Conclusions

We have reported the promising use of silk fibroin as sealant of biological tissues using a plasticised regenerated silk film. The addition of GNR to the 70:30 compositions, increases the stretchability, the water-capturing, and the mechanical contraction of the silk film. This enhanced the bursting pressure of anastomosed porcine intestine. Our study has shown that hybrid RS-GNR film could be used as bio-piezoelectric material for harvesting biomechanical movements. Such bio-films can be used for small to large diameter and can reduce the risks of the reconstructive surgical procedures. Additionally, silk fibroin can be fabricated with tuned mechanical plasticity, which enables its use in complex geometries if required.

## Supplementary Materials

The following are available online at https://www.mdpi.com/2079-4991/10/1/179/s1, Figure S1: FESEM images of the prepared samples; Figure S2: FTIR spectra with fitting procedure; Figure S3: Stress-strain curves of the prepared samples; Figure S4: The generated output short circuit current of piezoelectric devices fabricated with different RS and RS-GNR films. Figure S5: Bursting machine designed for this work. Videos of the contact angle measurements. Video S1: Video of contact angle measurement on 70:30 RS film. Video S2: Video of contact angle measurement on 70:30 RS-GNR film. Video S3: Video of contact angle measurement on 80:20 RS film.
