# Supplementary material for: Plasticised Regenerated Silk/Gold Nanorods Hybrids as Sealant and Bio-Piezoelectric Materials
Source: Nanomaterials (Basel). 2020 Jan 20;10(1):179. doi: 10.3390/nano10010179 (PMC7022986; doi:10.3390/nano10010179)
Supplement: Supplementary file 1 [file nanomaterials-10-00179-s001.zip › supplementary figures.pdf]

Supplementary material

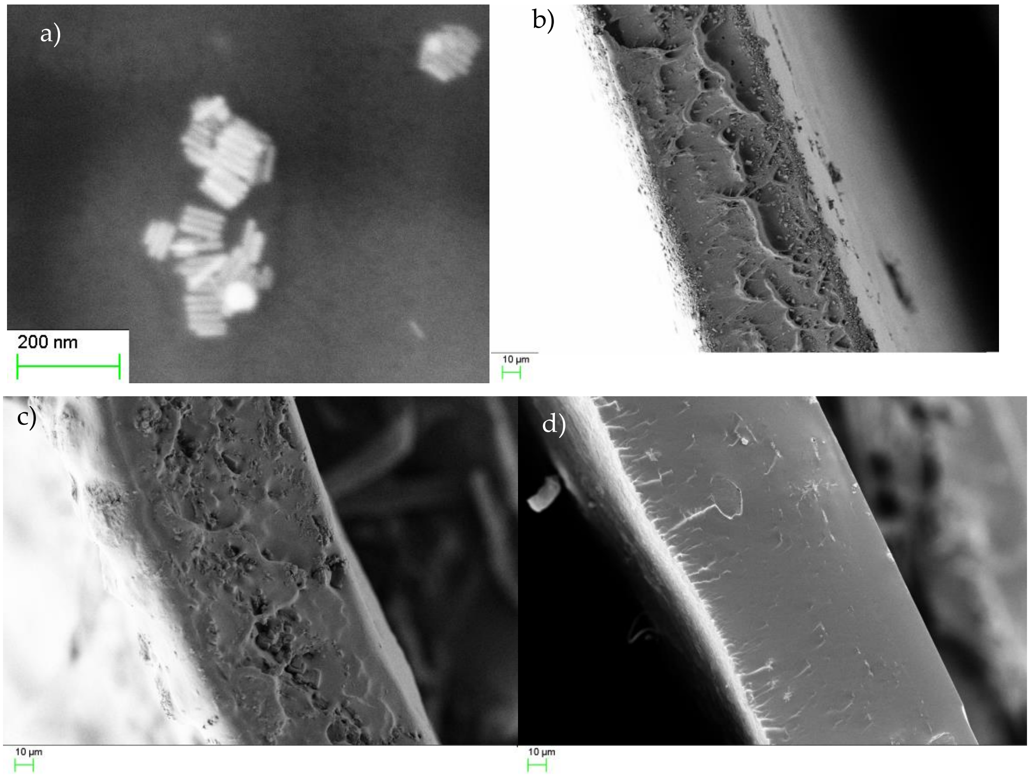

Figure S1. FESEM analysis showing the surface morphology of (a) GNR and cross-sections images of (b) 80:20 RS, (c) 80:20 RS-GNR and (d) 90:10 RS, respectively.

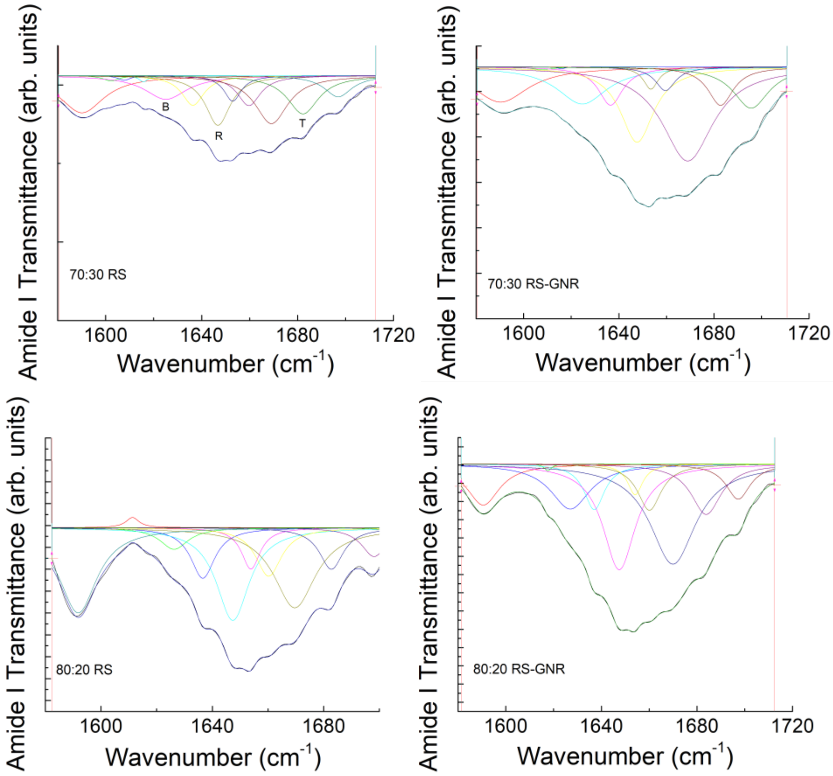

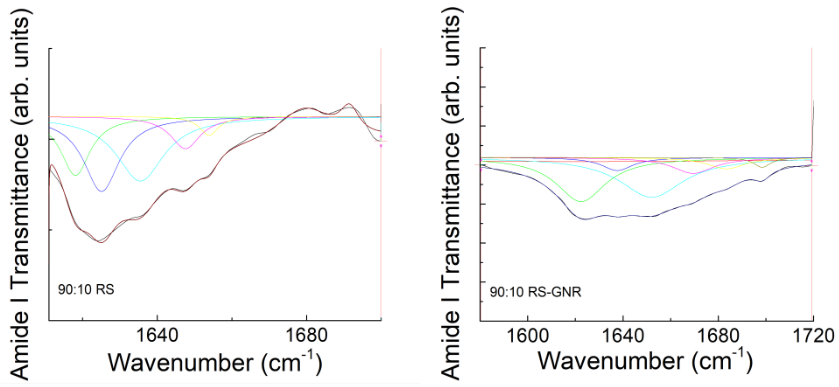

Figure S2. FTIR spectra of regenerated silk and RS/yeast films, respectively. The colored lines represent the components of the amide I band and are indicated as β-sheets (B), random coils (R), and turns (T).

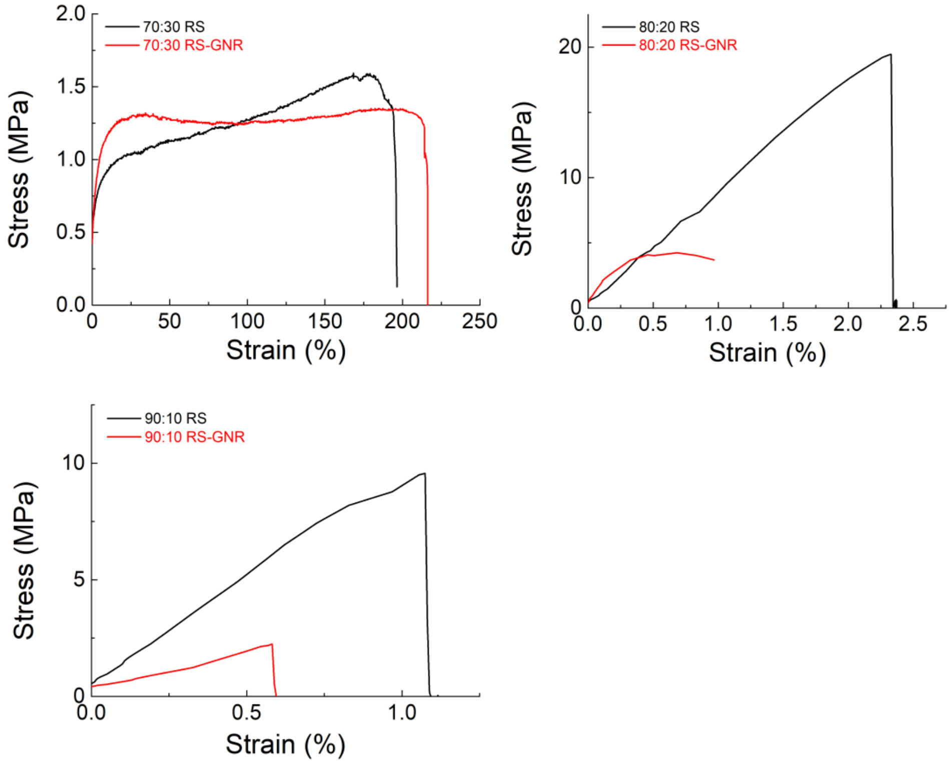

Figure S3. Stress-strain curves of RS and RS-GNR films.

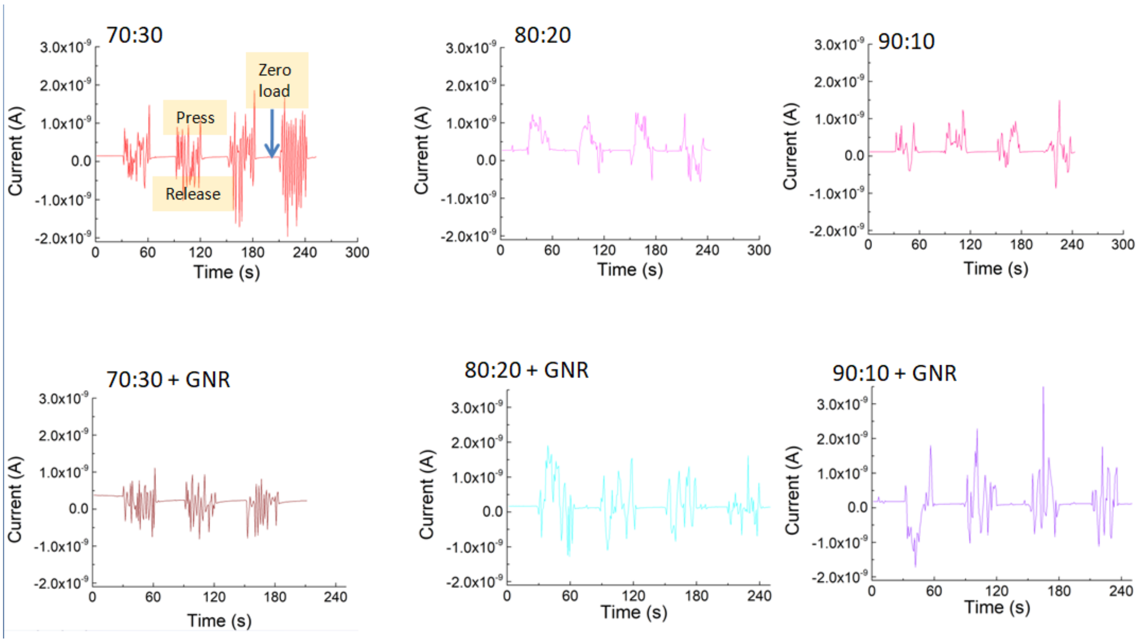

Figure S4. The generated output short circuit current of piezoelectric devices fabricated with different RS and RS-GNR films and subjected to repetitive loading and releasing pressure.

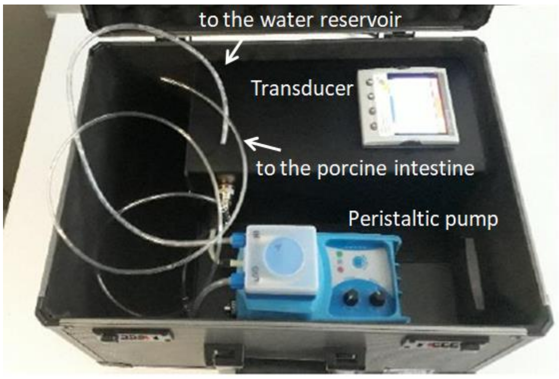

Figure S5. Bursting machine designed for this work.
